#  Rapid Extracellular Biosynthesis of Silver Nanoparticles by *Cunninghamella phaeospora *Culture Supernatant

**Published:** 2016

**Authors:** Mohamed Ghareib, Medhat Abu Tahon, Mona Mostafa Saif, Wafaa El-Sayed Abdallah

**Affiliations:** a *Department of Biological and Geological Sciences Faculty of Education, Ain Shams University, Roxy11757, Cairo, Egypt*; b *Department of Chemistry, Faculty of Education, Ain Shams University, Roxy 11757, Cairo, Egypt.*

**Keywords:** Characterization, *Cunnighamella phaeospora*, Rapid biosynthesis, Silver nanoparticles

## Abstract

The development of green approaches for the biosynthesis of silver nanoparticles (AgNPs) is of prime significance in the field of nanotechnology research. A fast and eco-friendly protocol for the biosynthesis of extracellular AgNPs using culture supernatant (CS) from the fungus *Cunninghamella phaeospora* was studied in this work. This CS was proved as a potential new source for the extracellular biosynthesis of AgNPs. The AgNPs were formed at 100 ^o^C and pH 9 within four min of contact between CS and 1mM silver nitrate (AgNO_3_) solution. Nitrate reductase (NR) was confirmed to play a pivotal role in the biosynthesis of AgNPs. The enzyme expressed its highest activity at 80 ^o^C and pH 9. At 100 ^o^C the enzyme retained 70% of its original activity for one hour. The half-life (T_1/2_) of the enzyme activity was calculated to be 1.55 h confirming its thermostability. The produced AgNPs were characterized by UV-Vis spectroscopy, high resolution-transmission electron microscope (HR-TEM) and x-ray diffraction (XRD). These NPs showed an absorption peak at 415 nm in UV-Vis spectrum corresponding to the plasmon resonance of AgNPs. Transmission electron micrographs revealed the production of monodispersed spherical NPs with average particle size 14 nm. XRD spectrum of the NPs confirmed the formation of metallic crystalline silver. It was also suggested that the aromatic amino acids play a role in the biosynthesis process. The current research provided an insight on the green biosynthesis of AgNPs including some mechanistic aspects using a new mycogenic source.

## Introduction

Production of metal nanoparticles (NPs) is one of the most important fields of nanotechnology. They are prepared by a variety of physical and chemical procedures but these require expensive, tedious and environmentally challenging techniques (1-2). The biological approaches attracted attention of many investigators in the last years aiming at development of cost effective, clean, non-toxic and environmentally benign procedures for synthesis of NPs (3). The biological systems of higher plants as well as microorganisms were used as reducing agents for metal ions to the irrespective NPs. Synthesis of NPs by the microorganisms interconnect nanotechnology and microbial biotechnology. NPs have been found to be produced by the action of different microbial species when exposed to solutions containing silver salts. Among the different microbes used for the synthesis of NPs fungi are efficient candidates for fabrication of metal NPs both intra-and extracellulary (4).These organisms are characterized by their fastidious growth, ease of handling and fabrication property (5). Moreover, their use holds promise for large scale metal NPs production as the enzymes secreted by fungi is an essential element for the biosynthesis of metal NPs (6). The extracellular procedure is more favorable than the intracellular in ease of downstream where the intracellular NPs require an additional step for their release from the biomass. Another advantage of the extracellular approach is the presence of high levels of secreted proteins and/or enzymes that not only stabilize the particles but also allows for an improved yield (7-8).

Silver NPs (AgNPs) have unique optical, electrical, and thermal properties that make it currently among the most widely used NPs in science. They have many applications in various areas especially in pharmaceutical and other biomedical applications (9-11).The extracellular biosynthesis of AgNPs by fungi was achieved by treating the silver salt i.e. AgNO_3_ with culture supernatant (CS)(5, 12-13), cell-free filtrate (CFF) obtained after incubating the preformed biomass with deionized water (14-17) or the preformed biomass directly (18-21). The initial approach of extracellular synthesis of Ag NPs by fungi was carried out by challenging a strain of *Verticillium* sp. with AgNO_3_ leading to silver reduction and formation of NPs (22). Other silver tolerant fungi like* Aspergillus niger* (8, 23), *Fusarium oxysporum* (24), *F. solani* (25), *Schizophylllum commune *(21) were recorded as suitable sources for these NPs**.**

A green mycological rapid technique for synthesis of AgNPs was elucidated in this work using the CS of the fungus *C. phaeospora* that not recorded in this regard before.

## Experimental

*Chemicals and glass wares*

All chemical used were of analytical grade. All reagent solutions were made with deionized water. AgNO_3_ was purchased from Sigma. The glass wares were washed with aqua regia (freshly mixed concentrated nitric acid and hydrochloric acid in a volume ratio of 1:3) to remove the traces of metal contaminant. 

*Organism and cultivation*

The fungus *C. phaeospora* Boedijn AUMC 8662 used throughout this work is a member of our lab collections. It was isolated from soil sample collected from the eastern province of Saudi Arabia and identified by Assiut University Mycological Center (AUMC) where it deposited with its accession number. The fungus was recorded previously (26) as an alkaliphilic organism. It was selected for this work in a preliminary screening of the fungi available in our lab on the basis of its rapid biosynthetic potentiality. Triplicate sets of 250 mL flasks, each containing 50 ml of the Czapek’s medium of the following composition (g/100 mL): sucrose, 3; NaNO_3_, 0.3; KH_2_PO_4_, 0.1; KCl, 0.05; MgSO_4_.7H_2_O, 0.5 and traces of FeSO_4_.5H_2_O were used. The flasks were sterilized for15 min at pressure of 15 libs /inch^2^, left to cool, initially adjusted to pH 9 and inoculated with 1ml of fungal spore suspension from 7-day-old-cultures contained 106spores. The cultures were incubated for 96 h at 30 °C on rotary shaker adjusted at150 rpm. By the end of the incubation period, the CS was separated from the biomass by filtration through Whatman filter paper No.1, centrifuged at 3000 rpm for 10 min to remove free cellular debris and used as the starting material for biosynthesis of NPs.

*Extracellular biosynthesis of AgNPs*

A triplicate set of 250 mL Erlenmeyer flasks each containing 90 mL of the CS and 10 mL of 10 mM AgNO_3 _in deionized water was added and mixed well to reach 1 mM final concentration of AgNO_3_. Simultaneously, appositive (CS) and negative controls (1 mM AgNO_3_) were also checked for comparison. All sets were kept under agitation (150 rpm) at 30 °C in the dark. Change in the colloidal solutions toward the brown color was taken as preliminary sign of AgNPs formation but their formation was confirmed spectrophotometrically.

*Optimization of the reaction conditions*

To study the effect of reaction temperature on formation of the NPs, triplicate sets of test tubes containing nine mL of CS were put separately in water bathes with different temperatures ranging 30 to 100 °C until their solutions acquire the investigated temperatures. 

To each tube one mL of 10 mM AgNO_3 _in deionized water was added, pH was adjusted to 9 in all cases and incubation completed as above. In the same way different tubes containing the above reaction mixture but incubated at100 °C were used for studying the effect of pH value in the range of 6-12. 

*Characterization of the synthesized NPs*

*UV-Visible spectroscopy analysis*

The bioreduction of Ag^+^ in the AgNO_3_ solution incubated with CS was monitored by measuring the UV-Vis spectrum of the reaction medium after diluting a small aliquot of the sample with deionized water. Absorption measurements were carried out at wave lengths from 300 to 800 nm using a double beam spectrophotometer (Metash UV-Vis, mode lUV-8500) at a resolution of 1nm. UV-Vis analysis of several weeks old sample was also achieved to check stability of the biosynthesized AgNPs.

For the additional characterization tests, the produced AgNPs were separated out by centrifugation at 17000 rpm for 15 min. The settled AgNPs were washed thrice with deionized water. When required the precipitated AgNPs either air-dried or redispersed in deionized water by ultrasonication (Chem Tec Ultrasonic Processor UP-500, SN: UH005-0076) to get rid of any uncoordinated biological molecules 

*High Resolution-Transmission Electron Microscopy (HR-TEM)*

The morphology and size of AgNPs were performed in nanotechnology and advanced material central lab (NAMCL), Agriculture Research Center, Cairo. 

For this purpose, an aliquot of the aqueous suspension of AgNPs was transferred onto a carbon coated copper grid. Samples were dried and kept under vacuum in desiccators before loading onto a specimen holder. The grid was then scanned using a Tecnai G20 (Fei, Netherland) HR-TEM operated at a voltage of 200 kV.

*X-ray diffraction (XRD)*

The XRD analysis was conducted on XPERT-PRO-PANalytical Powder Diffractometer (PANalytical B.V., Almelo, The Netherlands) using monochromatic Cu K α radiation (θ = 1.5406 Å) operating at a voltage of at 45 kV and a current of 30 mA at room temperature. The intensity data for the nanosilver powder were collected over a 2θ range of 4.01°–79.99°.

*Zeta potential measurement*

Zeta potential of AgNPs was evaluated using a Malvern Zetasizer Nanoseries Nano ZS (Malvern Instruments Ltd, Malvern, UK). An aqueous suspension of AgNPs was filtered through a 0.22 μM syringe driven filter unit before measurement. Data obtained were analyzed using Zetasizer software.

*Fluorescence emission spectrum*

Fluorescence emission spectrum of CS from *C. phaeospora* was achieved using Cary Eclipse FL 1006 M009 fluorescence spectrophotometer (Agilent Technologies, USA) at wavelengths 280 and 500 nm. The fluorescence measurement was run at room temperature (28 ^o^C) and the pH of the solution was adjusted to 9.

*Nitrate reductase (EC1.6.6.4) assay*

The procedure followed was that adopted by Harley (27). It depends upon potentiality of nitrate reductase (NR) in converting nitrate to nitrite. The enzyme activity was calculated based on the increase in nitrite over 60 min for the amount of sample 10 mL and expressed as U/mL. One unit of enzyme activity was tentatively defined as the amount of enzyme produce one µmol nitrite/h/mL.

## Results and discussion

*Biosynthesis of AgNPs*

Biosynthesis of AgNPs from AgNO_3_ is one of the most widely used procedures for the formation of silver colloids. This biosynthetic process was successfully performed by diverse of microorganisms including fungi. Most investigators reported the slow synthesis of AgNPs by fungi ranging from hours (5, 15-16, 18, 28) to days (14, 23, 29-30). However, AgNPs using *Aspergillus fumigatus* were obtained after 10 min (31) and this was the first report of rapid synthesis of AgNPs using fungi. The lengthy reaction is the major drawback of the biological synthesis approach that would have greater commercial viability if more rapidly synthesis of NPs is realized (32). 

**Figure 1:**
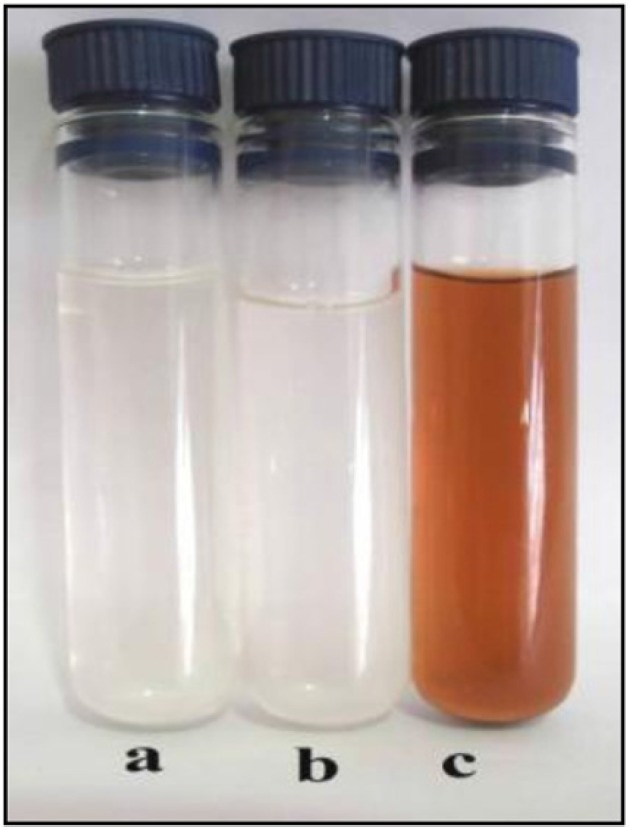
Color change of AgNo_3 _from colorless to brown on addition of CS © in comparisonwith the positive (b) and negative (a) controls.

**Figure 2:**
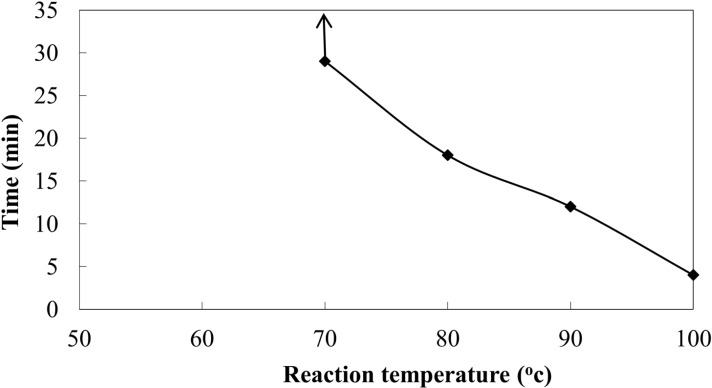
Effect of reaction temperature on time taken for biosynthesis of AgNPs using CS from *C.* *phaseospora*

**Figure 3:**
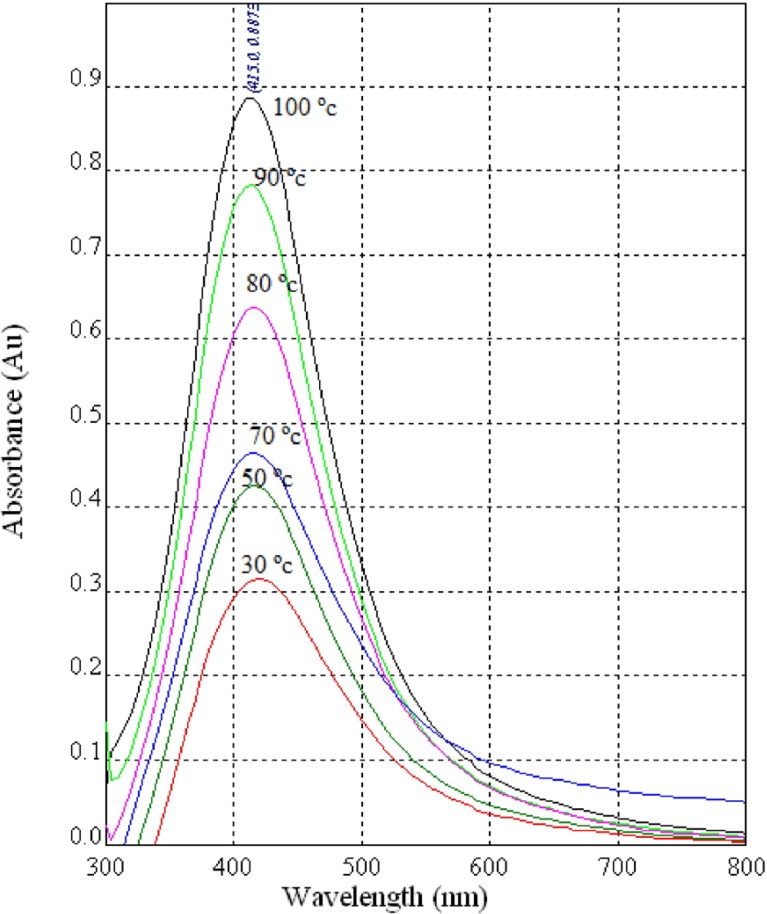
UV-Vis spectra of AgNPs synthesized using diluted CS (1:1) from the fungus *C.* *phaseospora* *at* *different* *reaction* *temperatures*

**Figure 4:**
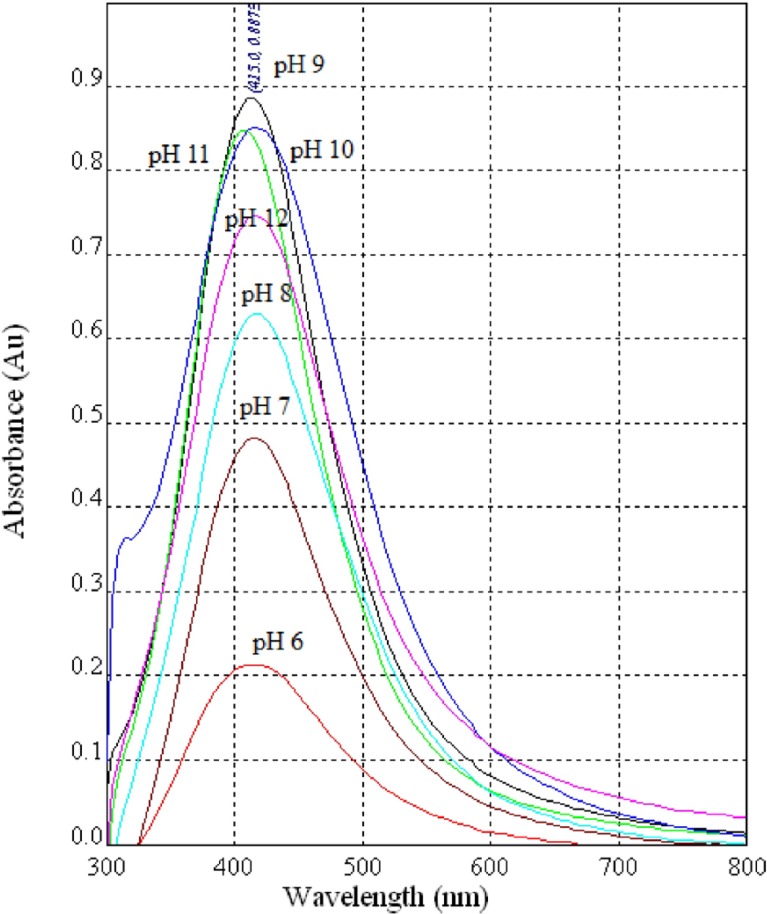
UV-Vis spectra of AgNPs synthesized using diluted CS (1:1) from the fungus *C.* *phaseospora* *at* *different* pH values

**Figure 5a:**
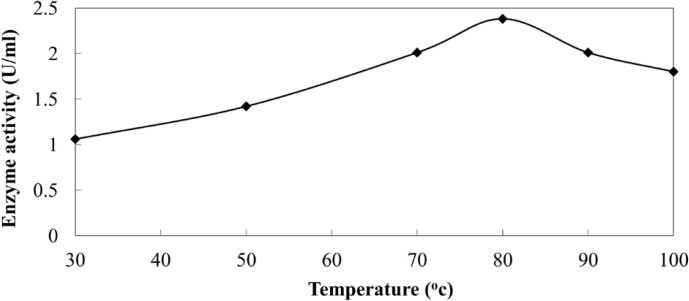
Activity of extracellular NR from *C.* *phaseospora* at different temperatures.

**Figure 5b:**
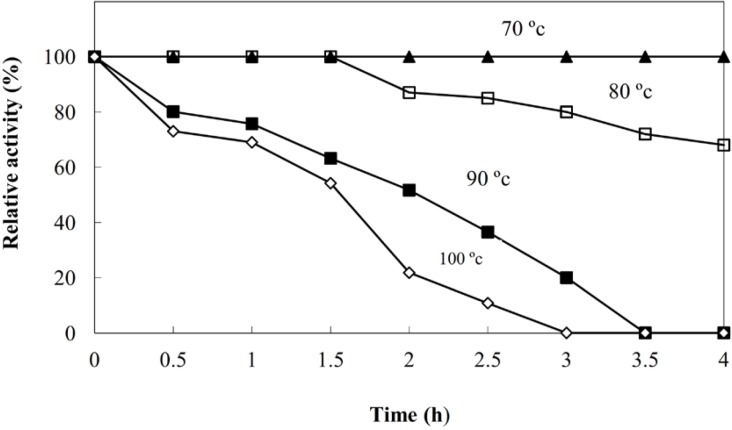
Termostability of the NR produced extracellularly in the CS of *C.* *phaseospora*

**Figure 6:**
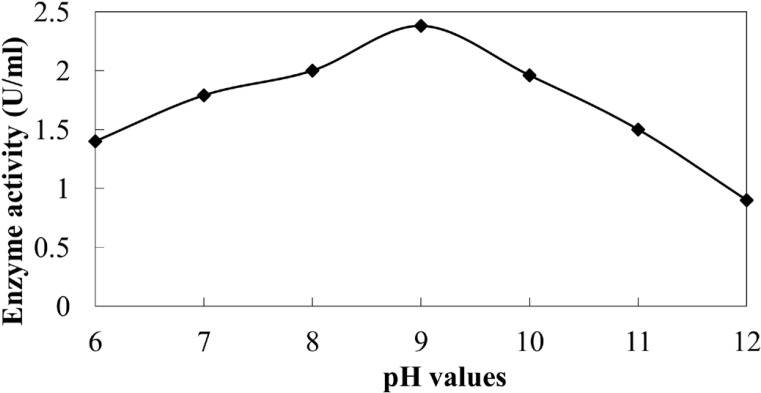
Activity of extracellular NR of *C.* *phaseospora* at different pH values.

**Figure 7a:**
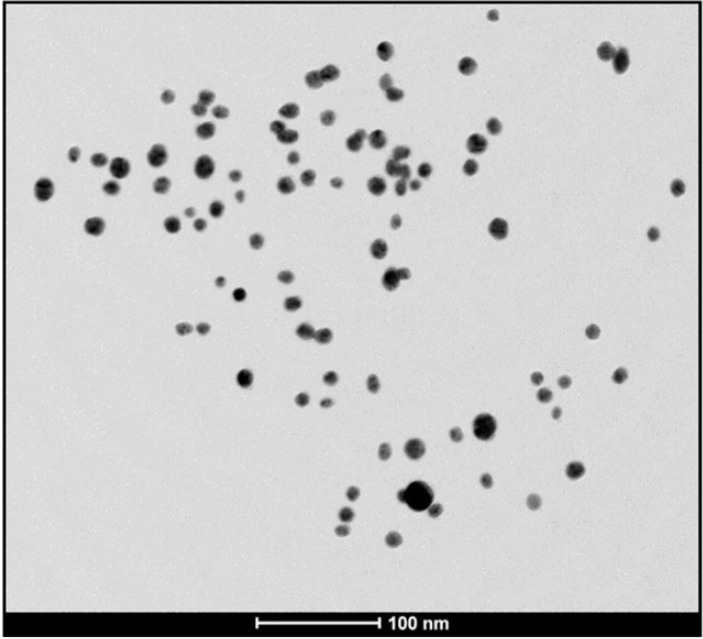
TEM image of the biosynthesized AgNPs using CS from *C. phaseospora *(Scale bar: 100nm

**Figure 7b:**
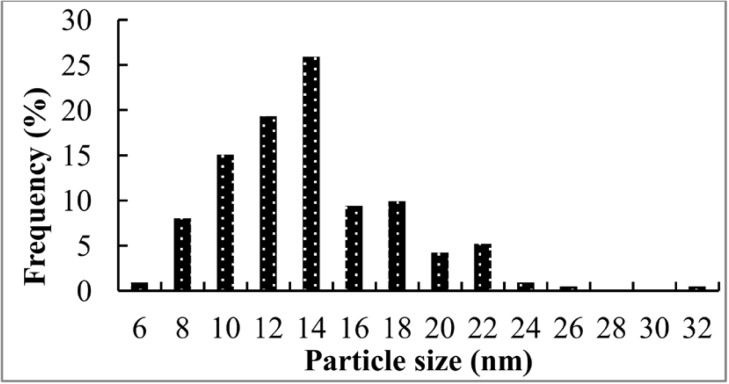
Particle size distribution of AgNPs from TEM analysis

**Figure 8:**
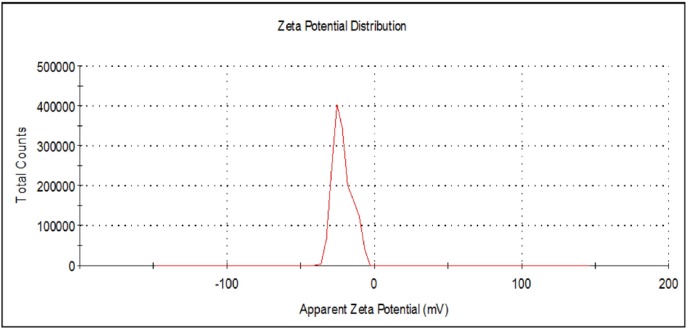
Zeta potential measurement of the biosynthesized AgNP.

**Figure 9:**
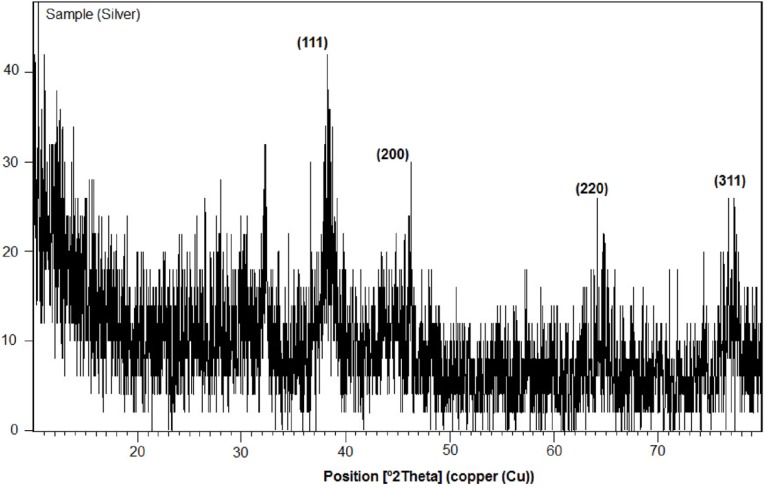
XRD pattern of AgNPs biosynthesized using CS from *C.* *phaseospora* (Labelled peaks are those corresponding to AgNPs

**Figure 10:**
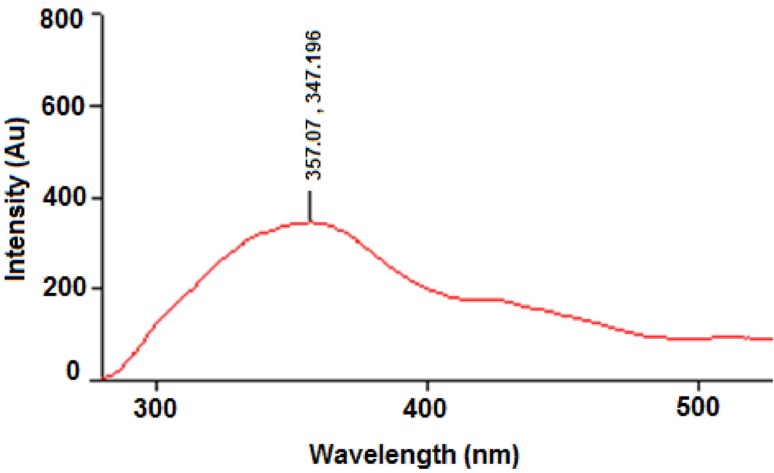
Fluorescence emission spectrum of CS from *C.* *phaseospora*

Formation of AgNPs by the aid of CS from the fungus *C. phaeospora* was preliminarily observed by the change in color from colorless to brown after 22 h of shaking AgNO_3_ with the fresh CS in the dark at 30 ^o^C (Figure 1). No change was observed in positive and negative controls under the same conditions. The brown color of silver colloid was attributed to specific surface Plasmon resonance (SPR) that occurs due to the collective oscillations of the conduction electrons confined to the NPs (5,18,33).

*Factors affecting the biosynthesis reaction*

*Effect of reaction temperature*

Certain factors affecting the reactions leading to the formation of AgNPs using CS from *C. phaeospora* were investigated. Reaction temperature produced a pronounced effect on both yield of the biosynthesized NPs and the time required for their biosynthesis. The rate of formation of the NPs was related to the reaction temperature and increased temperature levels allowed particle growth at a faster rate (Figure 2). At lower temperature such as 30 °C, complete reduction of silver ions took place about one day (exactly 22 h).This was previously accounted during the biosynthesis of gold NPs to the less activation energy and slow reduction activity of reducing agents present in the fungal solutions (34). At 100 ^o^C where there was an increase inactivation energy, AgNPs were produced within 4 min of reaction. This is a short time for biosynthesis of AgNPs from fungi and may be faster than that of the physical and chemical methods of NPs synthesis. Rapidity of formation is one of the key requirements for efficient production of the metal NPs which might be useful commercially for their scaling-up. This may led to the development of an easy bioprocess for synthesis of AgNPs.

Color patterns of the biosynthesized NPs at different reaction temperatures showed gradual increase in color intensity with increasing of temperature. UV-Vis spectral analysis of the NPs produced at different temperatures (Figure 3) reveals sharp SPR bands around 415 nm with a maximum at 100 ^o^C corresponds to the characteristic SPR band of spherical AgNPs (1,35).

*Impact of pH value*

Impact of pH value of the reaction mixture was also studied and found to be an important factor for achievement of the reduction process. Acidic conditions below pH 6 suppressed the formation of AgNPs where the process of biosynthesis was stopped completely at pH 5. On the other hand, the slight basic conditions (pH 6–8) enhanced the formation of AgNPs. Under alkaline conditions of pH 9-11, the enhancement effect was more pronounced reaching the maximum at pH 9 (Figure 4).This accord completely with those stated previously (36-38) where the biosynthesis of AgNPs enhanced as the pH increased towards alkaline region. A previous study show thatwhen the pH value increases, more competition occurs between protons and metal ions for negatively charged binding sites; therefore, a better synthesis of AgNPs at the alkaline pH has been recorded (36). So, it was reported that high temperature coupled with high ionic strength might be instrumental in enhancing the synthesis of AgNPs using CS from the bacterium* Pseudomonasa eruginosa *(39). The results of the present work accord completely with this explanation. The principle behind this process may be the activity of NR enzyme.

*Activity and stability of NR enzyme*

Activity of NR was measured in the CS, at a rate of 1.06 U/mL, suggesting its possible role in the biosynthesis process. The enzyme reduces nitrate to nitrite and thereby reducing Ag^+^ to Ag^0^ (40). To validate the possibility of enzyme action under these harsh conditions, both enzyme activity and stability were studied at different temperatures. The results revealed that the enzyme activity was greatly increased in the heated samples and reached the maximum at 80 ^o^C amounting to 2.38 U/mL (Figure 5a).

High thermostability of the enzyme was recorded (Figure 5b). The enzyme retained its full activity at 80 ^o^C for 1.5 h and below this temperature for at least 4 h. At 100 ^o^C, 70% of the original activity was recorded after one hour. T_1/2 _of the enzyme activity at 100 ^o^C was calculated to be 1.55 h. These results are in good agreement with those recorded for the NR from the hyperthermophilic archaeon *Pyrobaculum aerophilum *(41) and the bacterium* Enterobacter aerogenes* (42) and confirm the previous suggestion on the involvement of the NR enzyme in the biosynthesis of AgNPs. This clearly demonstrates that the reaction needs the enzyme NR and a great rate of the activation energy provided by the higher temperature.

The enzyme NR detected in the CS was proved to be an alkaline enzyme with optimum activity at pH 9 (Figure 6). A similar finding was previously obtained (43).Under the alkaline conditions, ability of enzymes responsible for the synthesis of metal NPs increases and this is an additional proves for the pivotal role of NR enzyme in reduction of silver ions to AgNPs.

*Characterization of the AgNPs*

Stability of the AgNPs biosynthesized at 100 ^o^C and pH 9 was monitored regularly for three months. It was found that these NPs were completely stable. Their surface plasmon band remains close to 415 nm suggesting that the particles are well dispersed in the aqueous solution. Transmission electron microgaph of the produced AgNPs (Figure 7a.) confirmed their stabilization where no sign of agglomeration was detected. 

These NPs were of an average size of 14 nm with size range from 6 to 32 nm (Figure 7b). Majority of the particles (88%) were less than 20 nm and the particles with sizes 10-14 nm were the predominant, representing 60% of the total content. In general, the NPs were spherical, monodispersed and uniformaly distributed. Monodispersity and stability are important and desired characteristics for commercial application of the NPs (31).

Additional prove for stability of the biosynthesized AgNPs was obtained from zeta potential measurement that was found to be -21.7 mV (Figure 8.) in comparison with -13.7 and -26.3 mV for AgNPs from *A. niger* (44) and* Penicillium sp*. (23), respectively. The negative value indicates electrostatic repulsion among the particles and thereby increasing the stability of the formulation (23,44).

Crystalline nature of the formed AgNPs was confirmed by their XRD analysis (Figure 9). The colloidal AgNPs showed four intense peaks of silver at 2*θ *= 38.126°, 44.313°, 64.463° and 77.422° that can be indexed at (111), (200), (220) and (311) facets which agree with the values reported for face centered cubic (fcc) silver nanocrystals (JCPDS card file NO. 4-783).

*Role of aromatic amino acids*

Application of the microbiological systems for the extracellular biosynthesis of AgNPs has already been reported earlier (24, 32). However, the exact agents leading to biosynthesis and stabilization of these NPs were yet to be elucidated. Some workers attributed stability of the NPs to the secretion of certain capping agents that is likely to be proteins (14, 32). Tryptophan in addition to tyrosine and phenylalanine are known as primarily responsible for the inherent fluorescence of proteins but tryptophan is the much more fluorescent. Presence of amino acids in the CS of the investigated fungus was confirmed by the ninhydrin positive reaction. The fluorescence emission spectrum of CS from *C. phaeospora* (Figure 10.) showed the presence of tryptophan (peak 347-357 nm) in this solution suggesting the role of aromatic amino acids as reducing and capping agents for AgNPs biosynthesized in this work. So we can safely state that the stability of the AgNPs may be due to presence of the capping proteins in their thermodynamically efficient state under the alkaline conditions.

## Conclusion

Utilization of culture supernatant from the fungus *C. phaeospora* for biosynthesis of silver nanoparticles represents a good alternative for the physical and chemical methods. This safe procedure was fast and produced stable nanoparticles with good characteristics. The biosynthesis process was affected largely by temperature and pH of the reaction mixture. Nitrate reductase and amino acids detected in the culture supernatant were suggested as important factors. Predominance of the alkaline conditions and high reaction temperatures were confirmed as essential requirements for the rapid biosynthesis of AgNPs. 
